# Exploring the Clinical and Psychosocial Impact of Genetic Diagnosis in Congenital Hearing Loss: A Comparative Study Between Syndromic and Non-Syndromic Conditions

**DOI:** 10.3390/children13070900

**Published:** 2026-07-06

**Authors:** Eva Orzan, Claudia Ceretta, Giulia Bresciani, Marta Fantoni, Paola Michieletto, Tiziana Di Cesare, Raffaella Marchi, Maria Teresa Bonati, Agnese Feresin

**Affiliations:** 1 Institute for Maternal and Child Health—IRRCS “Burlo Garofolo”, 34137 Trieste, Italy; eva.orzan@burlo.trieste.it (E.O.); claudia.ceretta@burlo.trieste.it (C.C.); marta.fantoni@burlo.trieste.it (M.F.); paola.michieletto@burlo.trieste.it (P.M.); tiziana.dicesare@burlo.trieste.it (T.D.C.); raffaella.marchi@burlo.trieste.it (R.M.); mariateresa.bonati@burlo.trieste.it (M.T.B.); 2 Independent Researcher, 33059 Fiumicello Villa Vicentina, Italy; feresin.agnese@gmail.com

**Keywords:** congenital hearing loss, genetic diagnosis, Usher syndrome, non-syndromic hearing loss, genetic counseling, psychosocial impact, parental empowerment, multidisciplinary care, follow-up

## Abstract

**Highlights:**

**What are the main findings?**

- A significantly longer time gap to genetic diagnosis was observed in the syndromic Usher cohort compared to the non-syndromic *GJB2* group.
- Exploratory analysis using our Italian-translated GCOS-24 version showed no significant differences in genetic empowerment between the two groups.

**What are the implications of the main findings?**

- The delayed genetic confirmation prolongs parental uncertainty and distress (“diagnostic odyssey”), underscoring the urgent need for earlier and faster genetic testing protocols in congenital hearing loss.
- Psychosocial adaptation depends heavily on the quality and continuity of clinical care rather than the diagnosis alone, highlighting the necessity of integrating longitudinal psychological support into multidisciplinary care.

**Abstract:**

**Background**: Genetic testing is increasingly part of the diagnostic pathway of congenital hearing loss (CHL), clarifying etiology and supporting clinical management. However, its psychosocial impact, especially differences between syndromic and non-syndromic conditions, remains underexplored. **Objectives**: This study evaluated the differential psychological impact of genetic diagnosis in syndromic versus non-syndromic pediatric patients, its relationship with clinical and rehabilitative variables, and the role of post-diagnostic psychological assessment. **Methods**: A cross-sectional post-diagnosis survey was conducted in families of children with genetically confirmed syndromic (Usher syndrome, *n* = 21) and non-syndromic (*GJB2*-related, *n* = 21) CHL; a total of 37 families responded. Parental empowerment was assessed using an Italian translated version of the Genetic Counseling Outcome Scale (GCOS-24). In an exploratory analysis, GCOS-24 items were grouped into three author-derived domains (understanding/awareness, emotional experience, and informational support) based on semantic content, not validated psychometrically. **Results**: No significant differences in GCOS-24 scores emerged between groups, nor in relation to clinical variables like hearing loss severity, auditory outcomes, or rehabilitative interventions. Genetic diagnosis occurred later in the syndromic group. Qualitative observations suggested parental empowerment varied with timing of diagnosis, clarity of information, and therapeutic alliance quality. **Conclusions**: Overall, these results highlight the importance of integrating psychological support and structured communication into clinical pathways to support families and patients in understanding and adapting to the diagnosis over time. Further longitudinal studies are needed to clarify the evolving psychosocial impact of genetic diagnosis in CHL.

## 1. Introduction

Congenital hearing loss (CHL) represents one of the most common sensory impairments in childhood, with a substantial proportion attributable to genetic etiologies [1,2]. Early detection strategies, including universal newborn hearing screening and pediatric surveillance programs, have been increasingly integrated into clinical practice [3]. Alongside these, advances in molecular diagnostics, particularly the widespread adoption of next-generation sequencing technologies such as whole-exome and whole-genome sequencing, have substantially enhanced the identification of causative variants in both syndromic and non-syndromic forms of hearing loss [1,3,4,5,6]. Taken together, these developments have contributed to shortening the so-called “diagnostic odyssey”, enabling earlier etiological clarification and facilitating more precise clinical management, including personalized therapeutic strategies, surveillance of associated comorbidities, and informed reproductive counseling for families [3,7,8].

In tandem with these advances, the clinical utility of genetic testing extends well beyond diagnostic yield alone. Growing attention has been directed toward understanding the broader psychosocial impact of receiving a genetic diagnosis, particularly in pediatric populations affected by rare diseases [9,10,11]. On one hand, a genetic diagnosis is often associated with positive expectations, including relief from uncertainty, improved access to tailored care pathways, and enhanced prognostic awareness. On the other hand, concerns persist regarding its potential psychological and emotional burden on families, including anxiety related to disease progression, guilt, stigma, and the implications for other family members [11,12,13]. These dual perspectives highlight the ethical, clinical, and rehabilitative complexities inherent to the integration of genetic testing into routine care.

Existing literature on the impact of genetic diagnosis has largely relied on service evaluation frameworks, typically assessing parental outcomes before and after genetic counseling interventions [3,11,14]. Studies in rare disease settings, for instance, have reported a reduction in parental state anxiety following diagnostic disclosure, suggesting a beneficial role of etiological clarification [11]. To evaluate this impact, several validated instruments have been developed; however, most are designed for oncology settings [15,16] or have limited global applicability [17], resulting in a limited availability of tools specifically tailored to rare genetic diseases such as syndromic and non-syndromic hearing loss [12,18]. The Genetic Counseling Outcome Scale (GCOS-24) is a notable exception, as it has been validated for assessing perceived empowerment in non-oncological populations undergoing genetic testing [14,19]. However, these approaches primarily assess the effectiveness of genetic services rather than the longitudinal impact of the diagnosis itself.

In the specific field of hereditary hearing loss, evidence remains limited and inconclusive [8,20]. Available studies have reported no significant differences in psychosocial outcomes between individuals who received a genetic diagnosis and those who did not [21]. Several factors may account for this null finding. First, most studies relied on relatively small cohorts, limiting statistical power to detect meaningful between-group differences. Second, the heterogeneity of genetic diagnoses themselves makes it difficult to draw uniform conclusions across patient groups when they are treated as a single category [11,19,21]. Furthermore, there is a notable lack of research comparing the impact of genetic diagnosis between syndromic and non-syndromic forms of CHL, despite their markedly different clinical trajectories, prognostic implications, and rehabilitative needs.

Importantly, most existing studies adopt a genetics-centered perspective, with limited integration of clinical variables relevant to long-term audiological and multidisciplinary follow-up. Yet, from the standpoint of the otolaryngologist and audiologist, understanding how a genetic diagnosis interacts with disease severity, therapeutic and rehabilitative interventions, auditory and language outcomes, and neurodevelopmental trajectories is crucial, as these factors are likely to shape the way families process and respond to diagnostic information. The subjective experience of families, encompassing emotional regulation, cognitive adaptation, and future-oriented expectations, may thus be influenced by these clinical contexts in ways that remain insufficiently explored by a purely genetics-centered approach [11].

The present study aims to address this gap by investigating the psychological impact of genetic diagnosis in a cohort of pediatric patients with syndromic and non-syndromic congenital hearing loss (NS-CHL and S-CHL, respectively), adopting a qualitative approach that integrates parental-reported outcomes with detailed clinical data. Particular attention is given to two autosomal recessive forms of isolated congenital hearing loss (CHL): *GJB2*-related hearing loss (*CX26*), which represents the most common cause of autosomal recessive non-syndromic hearing loss worldwide [13,14,15], and Usher syndrome, a syndromic condition. While *CX26* typically presents as an isolated, stable condition [22,23,24], Usher syndrome involves a more complex clinical trajectory due to progressive visual and vestibular impairment, as well as the heterogeneity that characterizes the three subtypes: Usher syndrome type I (USH1), type II (USH2), and type III (USH3) [25,26,27,28]; however, audiological outcomes can be favorable in both conditions when intervention is timely [25,26,27,29,30]. Rather than evaluating the genetic counseling process itself, this study focuses on the post-diagnostic phase to examine how families process and adapt to this information within ongoing clinical management. Specifically, the primary objective is to evaluate whether significant differences exist in the psychological impact of the diagnosis between the syndromic and non-syndromic groups. Secondarily, the study aims to assess how these emotional and cognitive responses correlate with the patients’ clinical and rehabilitative pathways (e.g., age at diagnosis, timeliness of intervention). By bridging genetic and audiological perspectives, this work ultimately highlights the crucial importance of assessing post-diagnostic psychological aspects in both groups. Understanding these dynamics is essential to move beyond a purely diagnostic function, informing long-term follow-up strategies and supporting the development of truly interdisciplinary care models that address the medical, psychological, and rehabilitative needs of these children and their families.

## 2. Materials and Methods

### 2.1. Study Design and Participants

This cross-sectional observational study was conducted at the Audiology Department of the Institute for Maternal and Child Health IRCCS “Burlo Garofolo” of Trieste, Italy, and approved by the Institutional Review Board IRCCS “Burlo Garofolo” (protocol number 17/23). The study was carried out in accordance with the principles of the Declaration of Helsinki.

Clinicians from the Audiology Department developed an online survey composed of two sections: (1) a section dedicated to the collection of information regarding the clinical and diagnostic pathway of patients, and (2) a section aimed at evaluating quality of life and psychological impact using the Italian translated version of validated questionnaires. In the present work, only the variables reported and the GCOS-24 instrument are considered.

The demographic and clinical variables investigated in this study, detailed below, were selected based on established literature [4,23,25] and institutional clinical practice. Specifically, variables assessed the timeliness of the clinical and rehabilitative pathway in alignment with the Joint Committee on Infant Hearing (JCIH) 2019 guidelines [31,32], while functional characteristics were based on WHO classification [33], adapted for the present study. The remaining parameters, such as specific time intervals, speech discrimination, and family participation scores [34], were included based on our daily clinical experience and expertise in managing pediatric hearing loss, ensuring a comprehensive overview of family’s adaptation post-diagnosis.

The survey was built-in using the LimeSurvey platform (LimeSurvey GmbH, Hamburg, Germany), which enabled distribution via email links to eligible participants, anonymous data collection, and the collection of informed consent for the use of data in medical research. Consent was mandatory to proceed with survey completion.

Before distribution, the survey was pilot tested in five families to assess clarity, comprehensibility, and ease of completion.

The survey was addressed to two groups of patients affected by congenital hearing loss, or to their parents in the case of minors: (1) patients with a genetically confirmed autosomal recessive syndromic hearing loss, i.e., Usher syndrome (USH-CHL), and (2) patients with genetically confirmed autosomal recessive non-syndromic hearing loss, i.e., DFNB1, *GJB2* Connexin 26 hearing loss (*GJB2*-CHL), serving as a comparison group.

The inclusion criteria were: clinical and genetic diagnosis of Usher syndrome for the first group; clinical and genetic diagnosis of *GJB2* non-syndromic hearing loss for the second group; follow-up at the Audiology Department of IRCCS “Burlo Garofolo”; and willingness to participate and adequate proficiency in Italian, as the survey was developed exclusively in this language. The exclusion criterion was the presence of cognitive disability or other significant comorbidities not included in Usher syndrome.

The protocol and follow-up regarding the management of patients with CHL is organized as follows at the Audiology Department of the IRCCS Burlo Garofolo: following universal newborn hearing screening (UNHS) or childhood auditory surveillance, all patients entered a structured diagnostic pathway integrating audiological, clinical, and genetic evaluation. After confirmation of CHL, first-line molecular testing is performed through targeted analysis of the *GJB2* gene, given its high prevalence in congenital non-syndromic hearing loss. In cases of negative *GJB2* results, patients underwent a stepwise re-evaluation process, including detailed clinical reassessment, longitudinal audiological follow-up, and targeted phenotyping aimed at identifying potential syndromic features. Broader genetic testing was then considered, including next-generation sequencing (NGS) approaches such as trio clinical exome sequencing.

The two groups of patients with USH-CHL or *GJB2*-CHL were retrospectively selected from clinical records and age-matched. Given that Usher syndrome is an autosomal recessive and rare genetic disease [25,26,27], all eligible patients with this condition currently monitored at the Audiology Department were invited to participate. To ensure comparability, the comparison group of *GJB2*-CHL patients was then selected based on the clinical characteristics of the syndromic cohort, resulting in a targeted sample of 21 subjects per group. If the participants were minors their questionnaires were completed by their parents, otherwise they were completed independently. The questionnaire was distributed to all eligible patients and remained online for three months (September–November 2025).

Text was translated and corrected using AI.

### 2.2. Patient’s Clinical Variables

**Figure 1:**
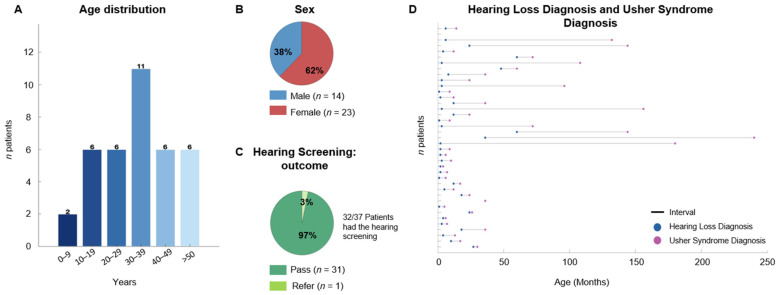
Demographic and audiological characteristics of the study cohort (*n* = 37). (**A**) Age distribution of participants across six age groups (years). (**B**) Sex distribution: 14 males (38%) and 23 females (62%). (**C**) Newborn hearing screening outcome: 32 out of 37 participants underwent the screening, of whom 31 (97%) received a “Refer” result and 1 (3%) a “Pass” result. (**D**) Individual timeline of hearing loss diagnosis (blue dots) and Usher syndrome genetic diagnosis (purple dots) for each participant. Horizontal lines indicate the interval between the two diagnoses.

Demographic data (age and sex) and information related to the diagnostic and therapeutic pathway were collected (Figure 1). Selected clinical variables were verified by cross-checking the survey responses with the corresponding data recorded in the patients’ medical records. The following variables were investigated:

(1) Degree of hearing loss, assessed by pure-tone average (PTA) as the mean unaided hearing threshold at 500, 1000, 2000, and 4000 Hz in the better ear, was categorized according to a WHO-based classification adapted for the present study as mild (20–34 dB HL), moderate (35–49 dB HL), moderately severe (50–64 dB HL), severe (65–79 dB HL), and profound (≥80 dB HL) [33];
(2) Usher syndrome genes involved with mutation profile; or *GJB2* mutation profile;
(3) If the neonatal hearing screening has been performed, and its outcome (“Refer” or “Pass”);
(4) Age at hearing loss identification expressed in months, i.e., the time at which the presence of sensorineural hearing loss was confirmed to the family;
(5) Current hearing aid (HA) use;
(6) Age at first HA fitting in months;
(7) Current cochlear implant (CI) use;
(8) Age at first cochlear implantation in months;
(9) Speech discrimination (SRT) in the best aided ear at the last audiometric evaluation prior to questionnaire completion, reflecting auditory-verbal discrimination ability as assessed by speech audiometry and expressed as the percentage of correctly recognized words at a conversational intensity level (65 dB HL);
(10) Current visual aid use;
(11) Family participation, defined as the quality of collaborative partnership between parents and early intervention professionals, reflecting mutual trust, involvement, and shared commitment to the child’s developmental goals [34]. This was rated on a 5-point scale (1 = limited participation; 5 = ideal participation), with higher scores indicating more effective family engagement, which is expected to positively influence the child’s rehabilitative progress;
(12) Family history of childhood hearing loss;
(13) Age at genetic diagnosis, defined as the age at which the genetic diagnosis was communicated to the family, expressed in months;
(14) Time interval between the age at identification of hearing loss (see item 4) and the age at communication of the genetic diagnosis (item 13);
(15) Time interval between the age at communication of the genetic diagnosis (item 13) and questionnaire completion.

### 2.3. Questionnaire

The Genetic Counseling Outcome Scale (GCOS-24) is a 24-item questionnaire that specifically measures empowerment, increasingly recognized as a key component of quality of life for individuals with genetic conditions. Currently, there is no clear consensus on how to evaluate psychosocial outcomes in the context of genetic counseling. The GCOS-24 was selected because it is one of the few validated tools designed to capture these aspects, especially in individuals with rare, non-oncological genetic disorders, providing a comprehensive assessment of perceived control, understanding, and ability to manage one’s condition. Its items are rated on a 7-point Likert scale (1 = strongly disagree; 7 = strongly agree), with total scores ranging from 24 to 168. No established cut-off values are available, and higher scores indicate greater empowerment. For minor pediatric patients, the questionnaire was completed exclusively by their parents, based on their own perceived family empowerment. This approach is highly appropriate since, in clinical genetics, parents of affected children may also be considered “patients”, as they remain at risk of having another affected child in future pregnancies [14,35]. Although the GCOS-24 is ideally administered both before and after genetic counseling, in this study, it was administered only after the genetic diagnosis.

Since the original questionnaire was available only in English, a translation into Italian was required for the target patient population. To ensure quality control, a forward-and-back translation procedure was implemented. The original English instrument was first translated into Italian and subsequently back-translated into English by two other independent translators blinded to the original text. The back-translated version was then compared with the original source to detect and rectify any inconsistencies, errors, or conceptual shifts, in accordance with the methodological framework proposed by Hall et al. (2018) [36].

The Genetic Counseling Outcome Scale (GCOS-24) was further explored through an author-derived, exploratory categorization of its items. Specifically, the questions were qualitatively grouped based on their semantic meaning into three exploratory domains: understanding/awareness, emotional experience, and informational support, with the exclusion of four items that were not retained for analysis, as they were considered repetitive and potentially confounding. This internal classification (each domain covering six, eight, and six items, respectively) was not based on established theoretical or psychometric criteria and was introduced to tentatively disentangle the multidimensional nature of the scale, which may limit its specificity for the present research objectives and should be interpreted as exploratory. The detailed distribution of the items within these three domains, along with the excluded items, are fully illustrated in Table 1. Subsequent analyses examined differences across these domains in relation to diagnosis (USH-CHL vs. *GJB2*-CHL) and degree of hearing loss (profound HL (>80 dB) vs. others), while the association between age at timing of genetic diagnosis (under 12 months vs. others) and outcomes was specifically explored for the emotional domain.

### 2.4. Statistical Analysis

Given the exploratory nature of the study and the limited sample size, only descriptive statistical analyses were performed. Continuous variables were summarized using the median and interquartile range (IQR), whereas categorical variables were reported as absolute frequencies and percentages. All descriptive analyses were conducted using Jamovi statistical software (version 2.6.44; The Jamovi Project, Sydney, Australia). Moreover, independent-samples *t*-tests were conducted to explore potential differences in GCOS-24 scores between the study groups.

### 2.5. Reliability Tests: McDonald’s Ω and Cronbach’s Alpha

We evaluated internal consistency across GCOS-24 questionnaire item responses using MATLAB (version R2023b; The MathWorks Inc., Natick, MA, USA). Prior to analyses, data quality was assessed by screening for missing values across all items; only one was identified and replaced using item mean imputation. Internal consistency reliability was estimated using Cronbach’s alpha (α) and McDonald’s omega (ω). Cronbach’s alpha was calculated from the item variances and the total score variance. McDonald’s omega total was estimated using a one-factor exploratory factor analysis based on principal axis factoring, from which factor loadings and unique variances were extracted.

## 3. Results

### 3.1. Cohort Description

The questionnaire was distributed to 42 eligible patients, including 21 with congenital hearing loss associated with Usher syndrome (USH-CHL) and 21 age-matched patients with non-syndromic CHL associated with a pathogenic mutation of the *GJB2* gene (*GJB2*-CHL). Overall, 37 participants (88.1%) completed the questionnaire, whereas five patients (11.9%) did not complete it or discontinued the survey, citing the sensitive nature of some of the topics addressed.

Among the 37 respondents, 19 (51.35%) had Usher syndrome and 18 (48.65%) had non-syndromic hearing loss. A total of 31 (83.78%) participants were minors, including 16 patients with USH-CHL and 15 with *GJB2*-CHL; the remaining six participants (16.22%) were affected adults (three in each group: USH-CHL and *GJB2*-CHL). Fourteen participants (37.84%) were male, and 23 (62.16%) were female. Participants’ year of birth ranged from 2001 to 2024.

The main characteristics of HL degree and genotype of the studied cohort are summarized in Table 2. The table reports hearing loss severity and genotype according to CHL type. Unaided hearing loss degrees are shown.

In the USH-CHL group, the most represented clinical subtype was USH2, diagnosed in 14/19 patients (73.69%), whereas 5/19 patients (26.31%) had USH1. Regarding the degree of hearing loss at identification (better ear), more than half of the patients (56.76%) presented profound hearing loss (>80 dB). This condition was particularly frequent in the *GJB2*-CHL group (88.88%). In contrast, patients with USH-CHL showed a more heterogeneous distribution, with a higher proportion of moderate-to-severe hearing loss; although, as expected, all five patients with USH-CHL type 1 presented with profound HL.

The main characteristics of the diagnostic and rehabilitative pathway of the patients included in the study are summarized in Table 3. Some additional variables are described narratively in the text and are therefore not presented in the tables.

Newborn hearing screening (NHS) was performed in the majority of the sample (32/37 patients, 86.49%), with similar percentages in the two groups (84.21% in patients with USH-CHL and 88.89% in patients with *GJB2*-CHL). Among the screened patients, the result was “refer” in 31 cases (96.88%), since only one patient presented a “pass” result.

The median age at hearing loss identification in the overall sample was 4 months (IQR 2–18), with comparable values in the two groups (USH-CHL: 4 months, IQR 3–18; *GJB2*-CHL: 4 months, IQR 2–16.5). The overall range varied from 1 to 60 months.

A history of hearing aid (HA) use was reported in 36/37 patients (97.3%). In the total sample, the median age at first HA fitting was 7.5 months (IQR 4–24), occurring earlier in *GJB2*-CHL than in USH-CHL (4 vs. 12 months). The median interval between hearing loss identification and HA fitting was 1 month (IQR 0–2.25), and appeared similar in the two groups.

At questionnaire completion, 16/37 patients (43.24%) were current HA users, including 12/19 (63.16%) in the USH-CHL group and 4/18 (22.22%) in the *GJB2*-CHL group. Current cochlear implant (CI) use was reported in 23/37 patients (62.16%), including 7/19 (36.84%) in the USH-CHL group and 16/18 (88.89%) in the *GJB2*-CHL group. HA and CI use were not mutually exclusive, as some patients used an HA on one side and a CI on the other. The median age at first cochlear implantation was 13 months (IQR 11–24) in the overall sample, occurring earlier in *GJB2*-CHL than in USH-CHL (12.5 months, IQR 10.75–21 vs 24 months, IQR 15–60). The median interval between hearing loss identification and cochlear implantation was 9 months (IQR 8–13.5; range 0–90) in the overall sample, and was longer in the USH-CHL group than in the *GJB2*-CHL group (16 months, IQR 12–29 vs 8.5 months, IQR 7.75–9.25, respectively).

In terms of speech recognition ability (SRT at 65 dB in the better ear with hearing aids/cochlear implantation), the majority of patients (33/37, 89.19%) showed scores ≥90%, with slightly higher percentages in the USH-CHL group (94.73%) compared with the *GJB2*-CHL group (83.33%).

Additional clinical features were evaluated in the entire sample to explore potential differences between groups. These variables included the use of visual aids, age at independent walking acquisition, and family participation data. In the overall sample, 20/37 patients (54.05%) reported the use of visual aids (e.g., prescription glasses, corrective lenses, or sunglasses prescribed for significant photosensitivity), whereas 17/37 patients (45.95%) did not use any visual aids. The use of visual aids was more frequent in the USH-CHL group, where 14/19 patients (73.68%) reported using them, compared with the *GJB2*-CHL group, in which only 6/18 patients (33.33%) used visual aids.

Family participation was distributed across categories 5–2 of the therapeutic alliance scale [23], with higher levels of participation prevailing in the overall cohort. Category 5, indicating ideal participation, was the most frequent level in the total sample, accounting for 16/37 patients (43.24%), followed by category 4 (good participation) in 10/37 (27.03%), category 3 (average participation) in 9/37 (24.32%), and category 2 (below-average participation) in 2/37 (5.41%) patients. In the USH-CHL group, family participation was more commonly distributed in the intermediate-to-high range, with category 4 being the most represented (7/19, 36.84%), followed by category 5 (6/19, 31.58%), category 3 (5/19, 26.32%), and category 2 (1/19, 5.26%). In contrast, the *GJB2*-CHL group showed a higher proportion of families in category 5 (10/18, 55.56%), followed by category 3 (4/18, 22.22%), category 4 (3/18, 16.67%), and category 2 (1/18, 5.56%). Overall, these findings suggest generally good family participation in both groups, with a greater concentration of ideal participation in *GJB2*-CHL and a more frequent distribution in the good participation range in USH-CHL.

Regarding walking development, delayed independent walking was defined as acquisition after 18 months of age [16,17,18]. Delayed walking is considered a common early indicator of USH-CHL type 1 and is typically associated with severe vestibular dysfunction present from birth. In the overall sample, the median age of independent walking acquisition was 12.5 months (IQR 11–18; min–max 8–42). When the two groups were analyzed separately, the median age was 12 months (IQR 11–18.5; min–max 8–42) in patients in the USH-CHL group and 13 months (IQR 11–15; min–max 10–20) in patients in the *GJB2*-CHL group. All patients with USH-CHL type 1 presented a delayed independent walking age, with a median of 24 months (IQR: 20–24).

A positive family history of congenital hearing loss was reported in 11/37 patients (29.73%), whereas 25/37 patients (67.57%) reported no family history; one patient was uncertain about the presence of other affected family members. A positive family history was more frequent in the USH-CHL group, where 8/19 patients (42.11%) reported affected relatives, compared with the *GJB2*-CHL group (3/18 patients, 16.67%).

The median age at genetic diagnosis in the overall sample was 24 months (IQR: 9–72), with notable differences between the two groups. In patients with USH-CHL, the genetic diagnosis occurred later (median 72 months, IQR 24–138) compared with patients with *GJB2*-CHL (median 8 months, IQR 6–15).

Similarly, the time interval between hearing loss identification and genetic diagnosis was longer in the USH-CHL group (median 28 months, IQR 12–112.5) than in the *GJB2*-CHL group (median 4 months, IQR 1.75–5.25). In the overall sample, this interval had a median duration of 8 months (IQR 4–48.5). The minimum observed interval was 0 months. This referred to a specific case of a 3-year-old child with a late diagnosis of profound hearing loss, in which genetic testing was performed urgently because the mother was pregnant and fetal risk assessment was required.

Conversely, the time between genetic diagnosis and questionnaire completion showed the opposite pattern, with longer intervals in the *GJB2*-CHL group (median 96.5 months, IQR 55–161.25) compared with the USH-CHL group (median 35 months, IQR 29–61).

### 3.2. Patient-Reported Genetic Counseling Outcome Scale-24 Items

The Genetic Counseling Outcome Scale (GCOS-24), a validated questionnaire designed to assess empowerment following genetic counseling and diagnosis, was administered in an Italian translated version, used for the purposes of this exploratory study, to *n* = 41 participants and then completed by *n* = 37 respondents. In contrast to its more typical use in the literature, the questionnaire was administered only after the communication of the genetic diagnosis and was therefore used in the present study as a cross-sectional comparative measure rather than as a pre-/post-counseling outcome.

In the overall sample, the median GCOS-24 score was 111 (IQR 105–116). Similar values were observed across the two groups, with median scores of 109 in the USH-CHL group and 114 in the *GJB2*-CHL group. We further explored whether GCOS-24 scores differed according to hearing loss severity (profound hearing loss, >80 dB, vs. all other degrees), age at hearing loss identification (≤6 months, in accordance with JCIH recommendations [31,32], vs. >6 months), speech recognition (100% vs. other values), visual aid use (yes vs. no), family participation (score of 5 vs. all other scores), and age at genetic diagnosis (<12 months vs. ≥12 months); when performing the independent-samples *t*-test, no statistically significant differences emerged in any of the comparisons performed (all *p* > 0.05). Median, IQR, and minimum–maximum values are reported in Table 4.

In addition, a secondary exploratory and unvalidated domain-based analysis of the GCOS-24 responses was conducted using the author-derived item classification described in the Materials and Methods section. Items were grouped into the understanding/awareness, emotional experience, and informational support domains, and compared between the USH-CHL and *GJB2*-CHL groups and according to hearing loss severity (>80 dB vs. all other degrees). For the emotional experience domain, an additional comparison was performed according to age at genetic diagnosis (<12 months vs. ≥12 months).

No differences emerged from any of these exploratory analyses. Given that the domain classification was not validated and was developed specifically for this study, these findings should be interpreted with caution and considered as hypothesis-generating only.

### 3.3. Reliability Analysis of the Questionnaire

The questionnaire demonstrated high internal consistency. Indeed, the reliability analysis yielded a Cronbach’s alpha of 0.92 and McDonald’s omega of 0.92. These results indicate a high degree of shared variance among items and strong scale reliability. Importantly, the agreement between our measures supports the interpretation of the questionnaire as measuring a coherent latent construct.

## 4. Discussion

By focusing on the parental experience of genetic diagnosis within the continuum of audiological and rehabilitative care, this study addresses a clinically relevant yet still underexplored dimension of congenital hearing loss (CHL) management. While previous research has primarily evaluated outcomes of genetic counseling services or short-term psychological responses [3,8], our approach situates genetic diagnosis within a longitudinal, clinically integrated framework.

This perspective highlights the importance of embedding genetic information into ongoing otolaryngological and audiological follow-up and supports structured interdisciplinary collaboration among genetics, audiology, and rehabilitation services [3,20]. Such integration is particularly relevant in pediatric settings, where families engage with healthcare systems over extended periods and where adaptation is a dynamic, evolving process.

The genetic and audiological characteristics of the cohort were consistent with the expected clinical profiles reported in the literature. In particular, the predominance of USH2 and the association of USH1 with profound hearing loss align with previous genotype–phenotype correlations [25,26,27]. Similarly, the high prevalence of profound hearing loss in the *GJB2*-CHL group is in line with well-established data on DFNB1-related hearing loss, which often presents as congenital, bilateral, and severe-to-profound.

The high rate of newborn hearing screening and the early age at diagnosis observed in both groups reflect the impact of universal newborn hearing screening programs, which have been described as a “silent revolution” in pediatric care [4]. In line with previous studies, early identification allowed timely access to rehabilitation—the “1-3-6” rule, or even better, the “1-2-3” rule—which is known to be a critical determinant in language and cognitive outcomes [31,32]. Although hearing-aid fitting and cochlear implantation occurred earlier in the *GJB2*-CHL group, these differences appear to be primarily driven by audiological phenotype rather than by differences in clinical management strategies. Similar patterns have been reported in the literature, where intervention timing is closely linked to the severity and stability of hearing loss rather than etiological classification per se.

A similar pattern was observed for cochlear implantation. In the present cohort, cochlear implants were more frequent in the *GJB2*-CHL group (88.89%), implantation occurred at an earlier age (12.5 months vs. 24 months in the USH-CHL group), and the interval between hearing loss diagnosis and first cochlear implantation was shorter (8.5 months vs. 16 months) than in the USH-CHL group. These findings should be interpreted cautiously, but they are clinically coherent with the overall distribution of hearing loss severity in the cohort and, more specifically, with the predominance of USH2 in the syndromic group. Accordingly, the observed differences in cochlear implant use appear to reflect the expected consequences of the underlying audiological phenotype rather than a fundamentally different rehabilitative strategy [25,26,27,37,38].

From a clinical perspective, these descriptive differences in timing deserve attention even in the absence of inferential claims. Early fitting of hearing aids and timely cochlear implantation may influence not only auditory outcomes, but also language acquisition, parental expectations, and the organization of long-term care. In this sense, the later rehabilitative trajectory observed in the USH-CHL group may represent an early divergence in clinical management that becomes particularly meaningful in a condition characterized by progressive multisensory involvement [18,25,28].

At the last audiological assessment, the median age was similar in the two groups, and most patients showed speech recognition scores ≥90% at 65 dB in the best aided ear. Overall, this finding suggests that the rehabilitative outcomes achieved in the cohort were generally satisfactory. The few cases with lower speech recognition scores appear to be clinically interpretable outliers rather than evidence of a generalized limitation of treatment efficacy. More specifically, performance below 90% was associated with factors directly related to rehabilitation adherence and timing: one patient with a 70% score refused hearing-aid use, another with the same score had recently undergone cochlear implantation and had not yet completed auditory training, and another had only one cochlear implant and refused contralateral implantation. In addition, one patient with a 75% score refused cochlear implantation and relied exclusively on hearing aids despite the severity of hearing loss.

The additional clinical features observed in the cohort further support the internal coherence of the phenotypic profile. Visual aid use was more frequent in the USH-CHL group (73.68% vs. 33.33%), consistent with the progressive visual involvement that characterizes the syndrome. Likewise, motor clumsiness was descriptively more common in patients with Usher syndrome, and all patients with USH1 showed delayed independent walking, with a median walking age of 24 months. These findings are clinically relevant because delayed walking and early motor difficulties may represent important extra-auditory clues to syndromic disease, particularly in Usher syndrome type 1, where vestibular dysfunction is often present from birth [25,26,27,39].

One of the most relevant findings of the present study concerns the genetic pathway. In fact, genetic diagnosis occurred descriptively much later in patients with Usher syndrome (72 months) than in those with *GJB2*-CHL (8 months), and the interval between hearing loss diagnosis and genetic diagnosis was also markedly longer in the USH-CHL group (28 months in the USH-CHL group vs. 4 months in the *GJB2*-CHL group). The marked delay in genetic diagnosis observed in the USH-CHL group reflects a well-recognized challenge in syndromic hearing loss, where the absence of overt extra-auditory features in early childhood may delay the suspicion of a syndromic condition. Similar diagnostic delays have been reported in previous studies, particularly for Usher syndrome type 2, where visual symptoms typically emerge later [28]. This finding is clinically important because, in CHL, molecular diagnosis is not limited to etiological clarification. Rather, it contributes to prognostic framing, follow-up planning, recurrence risk counseling, and recognition of associated clinical features that may emerge over time [1,8,11]. This is particularly true for Usher syndrome, in which genetic diagnosis may facilitate ophthalmological surveillance, support the interpretation of vestibular and developmental findings, and allow earlier discussion with families about the progressive and multisensory nature of the condition [25,26,27]. 

From a psychosocial perspective, this delay may correspond to a prolonged phase of “diagnostic ambiguity”, during which families interpret the condition as isolated hearing loss [11]. The transition to a syndromic diagnosis may therefore represent a critical re-framing moment, requiring substantial cognitive and emotional adaptation [28]. For this reason, delayed genetic definition in Usher syndrome may affect not only clinical management but also the family’s ability to understand the condition, anticipate future needs, and gradually adapt to a diagnosis involving hearing, vision, balance, and long-term autonomy. In this sense, genetic diagnosis represents not only a medical turning point, but also an important moment in the family’s cognitive and emotional reorganization [8,11,28,40].

The high internal consistency of the Italian translated version of the GCOS-24, as indicated by both Cronbach’s alpha and McDonald’s omega results, supports the reliability of the scale in this sample and suggests that the instrument measures a coherent latent construct.

In the present study, no statistically significant differences in GCOS-24 scores were observed between families of children with syndromic and non-syndromic CHL. The absence of significant differences in GCOS-24 scores between groups is consistent with previous studies in hearing loss populations, which have reported limited or no measurable differences in psychosocial outcomes following genetic diagnosis [14,21]. Similarly, studies using empowerment-based measures in genetic counseling contexts have shown that perceived empowerment may not directly reflect disease severity or clinical complexity [14]. While this finding may initially appear counterintuitive given the distinct clinical trajectories of these conditions, several factors should be considered when interpreting this result in our exploratory study. First, the relatively small sample size, inherent to the rarity of Usher syndrome, limits the statistical power to detect subtle differences. Second, the interval between genetic diagnosis and questionnaire completion was often long, particularly in the *GJB2*-CHL group. This temporal distance may have allowed emotional and cognitive processes to stabilize over time, leading to a convergence in perceived empowerment. Third, all patients were followed within a highly specialized audiological setting characterized by early diagnosis, structured rehabilitation, and long-term multidisciplinary care. This optimal clinical context may have mitigated potential psychosocial differences between groups. An additional key aspect concerns the clinical composition of the cohort, as most patients with Usher syndrome included in this study had not yet developed the full syndromic phenotype at the time of evaluation, presenting primarily with hearing loss. As a result, from the family’s perspective, the child’s condition may have been perceived as largely comparable to non-syndromic hearing loss. This phenotypic overlap may partly explain the lack of marked differences in psychosocial responses between the two groups.

In this context and in addition to previous evidence [14,21], our findings support the hypothesis that empowerment represents a multidimensional construct influenced not only by diagnostic information, but also by longitudinal care experiences, access to rehabilitation, and the quality of interaction with healthcare providers.

Additional insight can be gained from qualitative examination of selected individual cases, which helps contextualize the variability in GCOS-24 scores beyond group-level analysis. Qualitative inspection of individual responses revealed a small number of cases with particularly low GCOS-24 scores. Interestingly, the two lowest mean scores were observed in the *GJB2*-CHL group. In one case, the respondent appeared to have a limited understanding of the condition, together with persistent confusion and concern about their own future, despite profound deafness and unilateral cochlear implantation. In the second case, although the respondent expressed a generally positive view of the future, several neutral responses were given to items concerning knowledge of the child’s condition and its implications. These observations suggest that even when the diagnostic pathway appears relatively well defined, informational gaps and unresolved concerns may persist. In this sense, the present findings support the need for clear communication and structured support for families, not only at the moment of diagnosis, but throughout the longitudinal care pathway [11,20].

Within the Usher syndrome type 1 subgroup, only two patients had already developed early visual manifestations at the time of assessment. In both cases, the evolving multisensory phenotype appeared to coincide with more complex clinical trajectories, including challenges in adherence to rehabilitative recommendations and increased need of clinical support. In one case, involving a young adult patient, significant psychological vulnerability was reported, whereas in another adolescent patient, the rehabilitative pathway required sustained multidisciplinary engagement to achieve adherence to bilateral cochlear implantation. These observations suggest that the emergence of extra-auditory features may represent a critical phase in which empowerment is more fragile and requires targeted clinical attention.

Interestingly, this pattern contrasts with that observed in another patient with Usher syndrome type 1 who received an early genetic diagnosis within the first year of life. In this case, parental responses reflected a more structured understanding of the condition and a more proactive engagement with the care pathway. Similarly, in a younger patient with a later genetic diagnosis, responses suggested a more gradual process of adaptation, potentially influenced by the delayed integration of genetic information into the clinical narrative. Although anecdotal, these observations support the hypothesis that timing of diagnosis and clarity of information may shape how families construct meaning around the condition and engage with long-term care.

In addition, two cases characterized by lower ratings in items related to the therapeutic alliance [34] (scores of 2–3) highlighted the potential impact of relational factors on perceived empowerment. In these situations, reduced alignment between families and healthcare providers appeared to be associated with greater uncertainty in disease understanding and less consistent engagement with the rehabilitative process.

Taken together, these case-based observations reinforce the notion that parental empowerment is not solely determined by diagnostic category, but emerges from a dynamic interaction between clinical evolution, timing of information, and quality of the therapeutic relationship. They also suggest that individual trajectories may reveal critical points of vulnerability that are not captured by group-level comparisons alone.

Looking forward, these considerations gain further relevance in light of the progressive integration of genomic testing into newborn screening programs [1,8].

Our findings highlight the value of early etiological genetic diagnosis, particularly for conditions such as Usher syndrome, where it enables timely prognostic framing and anticipatory care. However, the observed variability in parental empowerment suggests that access to genetic information alone is not sufficient to support effective adaptation [11,20], but must be accompanied by continuous communication, disease awareness support, and strengthening of the therapeutic alliance [11,28].

In this context, the main challenge will not only be to diagnose earlier, but to support families in understanding and adapting to the diagnosis over time.

Our study raises the hypothesis that the impact of genetic diagnosis may influence long-term clinical outcomes indirectly through its effect on family engagement, adherence to rehabilitation, and follow-up compliance. Further longitudinal studies are warranted to explore these relationships, as well as to investigate how genetic diagnosis may shape reproductive decision-making and familial risk perception over time.

A major limitation of the present study lies in the timing of questionnaire administration. The GCOS-24 was administered only after genetic diagnosis, preventing assessment of changes over time. A pre- and post-diagnosis design would represent the methodological gold standard to capture the dynamic impact of genetic diagnosis on parental empowerment.

In addition to the timing of questionnaire administration, another limitation concerns the cross-sectional design, which does not allow assessment of causal relationships or temporal evolution of empowerment. Furthermore, the lack of data on prior psychological support may represent a potential confounding factor.

Furthermore, the GCOS-24, although widely used, may not fully capture the complexity of psychosocial adaptation in congenital hearing loss within a long-term, multidisciplinary care framework. The present findings also highlight the need for more integrated, longitudinal, and context-specific tools capable of dynamically assessing the interaction between clinical variables, family experience, and evolving care pathways.

Overall, our findings suggest that the quality, continuity, and timing of audiological and rehabilitative care may play a more decisive role in shaping parental experience than the specific genetic diagnosis itself.

From this perspective, genetic diagnosis should not be considered a discrete endpoint, but rather a dynamic component within a broader, longitudinal care process, in which therapeutic alliance, adherence, and clinical outcomes are central determinants of family adaptation. This integrated view may help guide the implementation of genomic medicine in real-world pediatric settings [8,11].

## 5. Conclusions

This post-diagnosis survey study involving families of children with USH-CHL and *GJB2*-CHL explored parental experiences following genetic diagnosis in children with syndromic (USH-CHL) and non-syndromic congenital hearing loss (*GJB2*-CHL) within an integrated audiological–genetic framework.

The qualitative analytical approach revealed no significant differences in perceived empowerment—dimensions concerning emotional regulation, cognitive processing, and hope—between groups. While exploratory in nature, this pattern may suggest that psychosocial outcomes may be influenced more by the quality and continuity of clinical care than by the specific etiological diagnosis, though this interpretation requires confirmation in larger, prospective studies.

The timing of genetic diagnosis, the clinical trajectory, and the emergence of syndromic features appear to play a relevant role in shaping family experience.

However, given that this study focused specifically on GJB2-related and Usher syndrome cohorts to ensure sample homogeneity, these findings should be interpreted with caution and cannot be automatically generalized to all etiologies of syndromic and non-syndromic hearing loss.

These preliminary findings support the need for early etiological clarification, consideration of family adherence, and therapeutic alliance within structured, multidisciplinary follow-up, as well as for longitudinal psychosocial support tailored to the evolving needs of patients and families.

Future research should adopt prospective designs and develop more comprehensive assessment tools to better capture the dynamic interaction between genetic diagnosis, clinical variables, and family adaptation over time.

## Figures and Tables

**Table 1:** Author-derived exploratory categorization of the Genetic Counseling Outcome Scale (GCOS-24) items into conceptual domains and inclusion status.

GCOS-24 Items ^1^	Domain	Status
1. I am clear in my own mind why I am attending the clinical genetics service.	/ ^2^	Excluded
2. I can explain what the condition means to people in my family who may need to know.	Understanding/Awareness	Included
3. I understand the impact of the condition on my child(ren)/any child I may have.	Understanding/Awareness	Included
4. When I think about the condition in my family, I get upset.	Emotional experience	Included
5. I don’t know where to go to get the medical help I/my family need (s).	Informational support	Included
6. I can see that good things have come from having this condition in my family.	Understanding/Awareness	Included
7. I can control how this condition affects my family.	Emotional experience	Included
8. I feel positive about the future.	Emotional experience	Included
9. I am able to cope with having this condition in my family.	Emotional experience	Included
10. I don’t know what could be gained from each of the options available to me.	Informational support	Included
11. Having this condition in my family makes me feel anxious.	Emotional experience	Included
12. I don’t know if this condition could affect my other relatives (brothers, sisters, aunts, uncles, cousins).	Understanding/Awareness	Included
13. In relation to the condition in my family, nothing I decide will change the future for my children/any children I might have.	Understanding/Awareness	Included
14. I understand the reasons why my doctor referred me to the clinical genetics service.	Informational support	Included
15. I know how to get the non-medical help I/my family need(s) (e.g., educational, financial, social support).	Informational support	Included
16. I can explain what the condition means to people outside my family who may need to know (e.g., teachers, social workers).	Informational support	Included
17. I don’t know what I can do to change how this condition affects me/my children.	Understanding/Awareness	Included
18. I don’t know who else in my family might be at risk for this condition.	Informational support	Included
19. I am hopeful that my children can look forward to a rewarding family life.	Emotional experience	Included
20. I am able to make plans for the future.	/ ^2^	Excluded
21. I feel guilty because I (might have) passed this condition on to my children.	Emotional experience	Included
22. I am powerless to do anything about this condition in my family.	Emotional experience	Included
23. I understand what concerns brought me to the clinical genetics service.	/ ^2^	Excluded
24. I can make decisions about the condition that may change my child(ren)’s future/the future of any child(ren) I may have.	/ ^2^	Excluded

^1^ Items are presented in the original English version; the Italian version administered to the participants, used for the purposes of this exploratory study, is available from the corresponding author upon request. ^2^ Excluded items (Items 1, 20, 23, and 24) were removed from the domain sub-analysis due to semantic redundancy.

**Table 2:** Hearing loss degree and genotype of the studied cohorts.

Type of CHL		Degree of HL ^1^	Gene (*n*) ^2^	Genotype (*n*) ^7^
USH- CHL ^5^ (*n* = 19)	USH1 ^3^ (*n* = 5)	Profound HL	*MYO7A* (1)	c.3719G>A/c.6028G>A (1);
		Profound HL	*USH1C* (1)	c.711delT/c.711delT (1);
		Profound HL	*CDH23* (3)	c.3646_3647delCT/c.4562A>G (1); c.9433C>T/c.5712G>A (1); c.5985C>A/c.5985C>A (1).
	USH2 ^4^ (*n* = 14)	Mild HL	*USH2A* (11)	c.13392G>A/c.232T>G (1);
		Moderate HL		Homozygous deletion involving exons 5–10 (1); c.11864G>A/c.2299delG (1); c.2276G>T/c.11864G>A (1); c.9270C>A/c.5189_5199delATATGTTTCAT (1); c.11864G>A/c.11864G>A (1);
		Moderately severe HL		c.11864G>A/c.67056708delAACT (2); c.1876C>A/c.9270C>A (1); c.11864G>A/c.11864G>A (1); c.1055C>T/c.1055C>T (1);
		Moderate HL	*ADGRV1* (3)	c.2127_2137del/p.Asn4558fs (1);
		Moderately severe HL		c.4378G>A/c.13655dupT (1); c.13655dupT/c.9447-1G>A (1).
*GJB2*-CHL ^6^ (*n* = 18)		Moderate HL	*GJB2* (18)	35delG/c.269T>C (1);
		Moderately severe HL		c.71G>A/c.71G>A (1);
		Profound HL		35delG/35delG (14); 35delG/c.139G>T (1); 35delG/c.283G>A (1).

^1^ HL = Hearing loss; ^2^ *n* = sample size. Numbers in parentheses within the ‘Gene’ column indicate the number of individuals with that specific gene; ^3^ USH1 = Usher syndrome type 1; ^4^ USH2 = Usher syndrome type 2. ^5^ USH-CHL = Usher syndrome-related congenital hearing loss; ^6^ *GJB2*-CHL = *GJB2*-related non-syndromic congenital hearing loss. ^7^ Pathogenic and likely pathogenic variants for each gene are described according to the reference NM. Compound heterozygous and homozygous genotypes are indicated as variant1/variant2 and variant1/variant1, respectively. Segregation analysis confirmed variants in trans when applicable. Numbers in parentheses within the ‘Genotype’ column indicate the number of individuals with that specific genotype.

**Table 3:** Diagnostic and follow-up characteristics of patients in the study cohort.

Variable		Total Sample	USH-CHL ^8^	*GJB2*-CHL ^9^
NHS ^1^ performed	Yes	32/37 (86.49%) 5/37 (13.51%)	16/19 (84.21%) 3/19 (15.79%)	16/18 (88.89%) 2/18 (11.11%)
	No			
NHS ^1^ outcome	Refer	31/32 (96.88%)	15/16 (93.75%)	16/16 (100.0%)
	Pass	1/32 (3.13%)	1/16 (6.25%)	0/16 (0.0%)
Age at CHL ^2^ identification (months)	Median (IQR) ^3^	4 (2–18)	4 (3–18)	4 (2–16.5)
	Min–Max	1–60	1–60	1–36
Current HA ^4^ use	Yes	16/37 (43.24%)	12/19 (63.16%)	4/18 (22.22%)
	No	21/37 (56.76%)	7/19 (36.84%)	14/18 (77.78%)
Age at first HA fitting (months)	Median (IQR) ^3^	7.5 (4–24)	12 (6–33)	4 (3–17)
	Min–Max	1–60	3–60	1–36
CI ^5^ use (uni ^6^/bilateral)	Yes	23/37 (62.16%)	7/19 (36.84%)	16/18 (88.89%)
	No	14/37 (37.84%)	12/19 (63.16%)	2/18 (11.11%)
Age at first CI (months)	Median (IQR) ^3^	13 (11–24)	24 (15–60)	12.5 (10.75–21)
	Min–Max	9–96	10–96	9–36
Speech discrimination (SRT ^7^ 65 dB, best aided ear)	<90%	4/37 (10.81%)	1/19 (5.26%)	3/18 (16.67%)
	≥90%	33/37 (89.19%)	18/19 (94.74%)	15/18 (83.33%)
Visual aid use	Yes	20/37 (54.05%)	14/19 (73.68%)	6/18 (33.33%)
	No	17/37 (45.95%)	5/19 (26.32%)	12/18 (66.67%)
Family participation score	4–5	26/37 (70.27%)	13/19 (68.42%)	13/18 (72.22%)
	2–3	11/37 (29.73%)	16/19 (31.58%)	5/18 (27.78%)
Family history for CHL	Yes No Do not recall	11/37 (29.73%) 25/37 (67.57%)	8/19 (42.11%) 11/19 (57.89%)	3/18 (16.67%) 14/18 (77.78%)
		1/37 (2.7%)	0/19 (0.0%)	1/18 (5.56%)
Age at genetic diagnosis (months)	Median (IQR) ^3^	24 (9–72)	72 (24–138)	11 (6.25–22.25)
	Min–Max	4–240	9–240	4–36
CHL identification–genetic diagnosis time interval (months)	Median (IQR) ^3^	8 (5–28)	28 (12–112.5)	5 (3.25–7)
	Min–Max	0–204	8–204	0–18
Genetic diagnosis–survey completion interval (months)	Median (IQR)	60 (31–91)	35 (29–61)	96.5 (52–160)
	Min–Max	7–274	10–90	7–274

All variables are named as in the Materials and Methods section. ^1^ NHS = neonatal hearing screening; ^2^ CHL = congenital hearing loss; ^3^ IQR = interquartile range, data are presented as median (interquartile range); ^4^ HA = hearing aid; ^5^ CI = cochlear implant; ^6^ Uni = unilateral; ^7^ SRT = speech reception threshold. ^8^ USH-CHL = Usher syndrome-related congenital hearing loss; ^9^ *GJB2*-CHL = *GJB2*-related non-syndromic congenital hearing loss.

**Table 4:** GCOS-24 scores and descriptive comparisons across study groups.

Variables		GCOS-24 ^1^ score		
		Median	IQR ^4^	Min–Max
CHL ^2^ type	Total sample	111	105–116	84–135
	USH-CHL ^6^	109	104–115.5	98–135
	*GJB2*-CHL ^7^	114	106.25–119.75	84–134
Degree of HL ^3^	Mild to severe HL ^3^	109	104–115.25	98–135
	Profound HL ^3^	115	106–118	84–134
Age at HL ^3^ identification	≤6 months	110	105.25–115	84–124
	>6 months	113	102.5–122.5	95–135
Speech discrimination (SRT) ^5^	<100%	109	107.5–115.5	84–134
	100%	112.5	104.25–116.75	95–135
Visual aid use	Yes	111.5	105.75–118.75	84–135
	No	111	104–115	95–134
Family participation score	<5	113	104–116	84–134
	5	109	105–118.75	95–135
Age at genetic diagnosis	≤12 months	112	106–115.75	104–124
	>12 months	111	102.5–116.5	84–135

All variables are classified as in the Materials and Methods section. ^1^ GCOS-24 **=** Genetic Counseling Outcome Scale; ^2^ CHL = congenital hearing loss; ^3^ HL = hearing loss; ^4^ IQR = interquartile range; ^5^ SRT = speech reception threshold; ^6^ USH-CHL = Usher syndrome-related congenital hearing loss; ^7^ *GJB2*-CHL = *GJB2*-related non-syndromic congenital hearing loss. No statistically significant differences emerged in any of the comparisons performed (all *p* > 0.05).

## Data Availability

The original contributions presented in this study are included in the article. Further inquiries can be directed to the corresponding author.
